# Flypaper effect assessment methods in the expansion of regional autonomy

**DOI:** 10.1016/j.mex.2021.101387

**Published:** 2021-05-15

**Authors:** Abdul Hafiz Tanjung, Sazilah Salam, Jack Febrian Rusdi, Yana Ermawati, Ira Novianty, Raden Budi Hendaris, Yeti Apriliawati

**Affiliations:** a Jurusan Akuntansi, Fakultas Ekonomi, Universitas Nasional PASIM, Bandung, Indonesia; b Web Science Institute, School of Electronics and Computer Science, Faculty of Engineering and Physical Sciences, University of Southampton, UK; c Department of Interactive Media, Faculty of Information and Communication Technology, Universiti Teknikal Malaysia Melaka (UTeM), Malaysia; d Informatics Engineering, Sekolah Tinggi Teknologi Bandung, Bandung, Indonesia; e Jurusan Akuntansi, Fakultas Ekonomi dan Bisnis, Universitas Yapis Papua, Papua, Indonesia; f Jurusan Akuntansi, Politeknik Negeri Bandung, Bandung, Indonesia; g Jurusan Akuntansi, Fakultas Ekonomi dan Bisnis, Universitas Jenderal Ahmad Yani, Cimahi, Indonesia; 8 Program Studi D4 Akuntansi Manajemen Pemerintahan. Politeknik Negeri Bandung, Bandung, Indonesia

**Keywords:** Autonomous regions, Local government expenditure, General allocation fund, Original local government revenue, Degree of fiscal otonomy, Random Effect Model, Pooled Least Square, Fixed Effect model, Chow test, Lagrange Multiplier, Chow Test, Lagrange multiplier, Hausman Test, Koenker-bassett test

## Abstract

Flypaper Effect is a public finance term that indicates a government grant given to recipient cities increases the local community spending level more than an increase in local income of equivalent size. This paper analyzed the Flypaper Effect Assessment Method in the Expansion of Regional Autonomy. It employed 210 New Autonomous Regions (NARs) in Indonesia during 1999–2021 as a case study, where Indonesia became the country with the highest number of new autonomies in the world. Panel Data Regression was utilized to determine the Flypaper Effect. Flypaper Effect analysis was carried out using the BLUE model selection method. The selected models in this study were Pooled Least Square (PLS), Fixed Effect Model (FEM), and Random Effect Model (REM). Several tests, such as Chow Test, Lagrange Multiplier Test, and Hausman Test, were conducted. Furthermore, the procedures to get the data in BLUE were carried out, such as Heteroscedasticity and Autocorrelation Test. Koenker-Bassett test was used for ascertaining Heterocedascity.

• Panel Data Regression is used as a method to determine the Flypaper Effect in the autonomous region.
• Each stage in this method is discussed with a calculation/process example.
• The method utilized in this paper is recommended to determine the Flypaper Effect of New Autonomous Regions (NARs) for various parties.

## Graphical abstract

**Figure:**
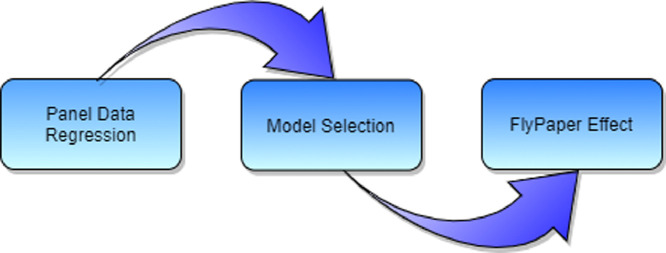

Specifications table

**Table:** 

Subject Area:	Economics and Finance
More specific subject area:	Flypaper Effect
Method name:	Flypaper Effect Assessment Methods in the Expansion of Regional Autonomy
Name and reference of original method:	Panel Data Regression Y. Y. Tesfay, “Modified panel data regression model and its applications to the airline industry: Modeling the load factor of Europe North and Europe Mid Atlantic flights,” Journal of Traffic and Transportation Engineering (English Edition), vol. 3, no. 4, pp. 283–295, Aug. (2016), doi:10.1016/j.jtte.2016.01.006.
Resource availability:	J. F. Rusdi, “Data Panel Regression Flypaper Effect in New Regional Autonomy,” Mendeley Data, V1., (2021), doi:10.17632/7fvk78fgv4.1.

## Method details

Regional autonomy is one of the ways for a government to provide independence for its regions, including the financial matter. The government expects the regional autonomy to be able to optimally finance its own regional expenditures and minimize the central government's budget [1].

The establishment of the New Autonomous Regions (NAR) occurs in almost all countries including Indonesia. An autonomous region is a part of a country with a degree of autonomy or independence from outside authorities. In Indonesia, the NAR establishment has increased sharply [2], especially since the enforcement of Law No. 22 of 1999. Hence, from 1999 until 2014, 210 NAR were inaugurated by the Indonesian government. There are 514 autonomous regions in Indonesia consisting of 416 districts and 98 cities. The new autonomous regions’ high growth makes Indonesia a country with the highest NAR growth in the world [3]. Besides, from 2014 until early 2021, there were at least 314 submissions to the government [4]. In 2014, the establishment of NAR was suspended because of a growing number of General Allocation Funds (GAFs) being allocated to all autonomous regions, which is closely related to the occurrence of the Flypaper Effect in the autonomous region [5,6].

The Flypaper Effect is a public-finance term which indicates that a government grant given to recipient cities increases the local community spending level more than an increase in local income of an equivalent size. Flypaper Effect is a public finance principle which suggests that the government grants to the recipient cities increase more than an equal increase in local revenue, the level of local public spending [7].

Panel Data Regression can be utilized to study the Flypaper Effect. However, based on search results through Science Direct, the Flypaper Effect method to evaluate NAR has never been studied [8]. Hence, the researchers proposed the Flypaper Effect test method on NAR. This method relates to a case study of NAR expansion in Indonesia.

This study uses Panel Data Regression models [9], a combination of cross-section (NAR) and time series (years of data understudy). Furthermore, three potential estimation models of Panel Data Regression were used namely Pooled Least Square (PLS) [10], Fixed Effect Model (FEM) [11], and Random Effect Model (REM) [12]. In the process, the system was to choose one of these models to determine the Flypaper Effect. FEM and PLS are models that often meet the Best Linear Unbiased Estimator (BLUE) [13].

**Fig. 1:**
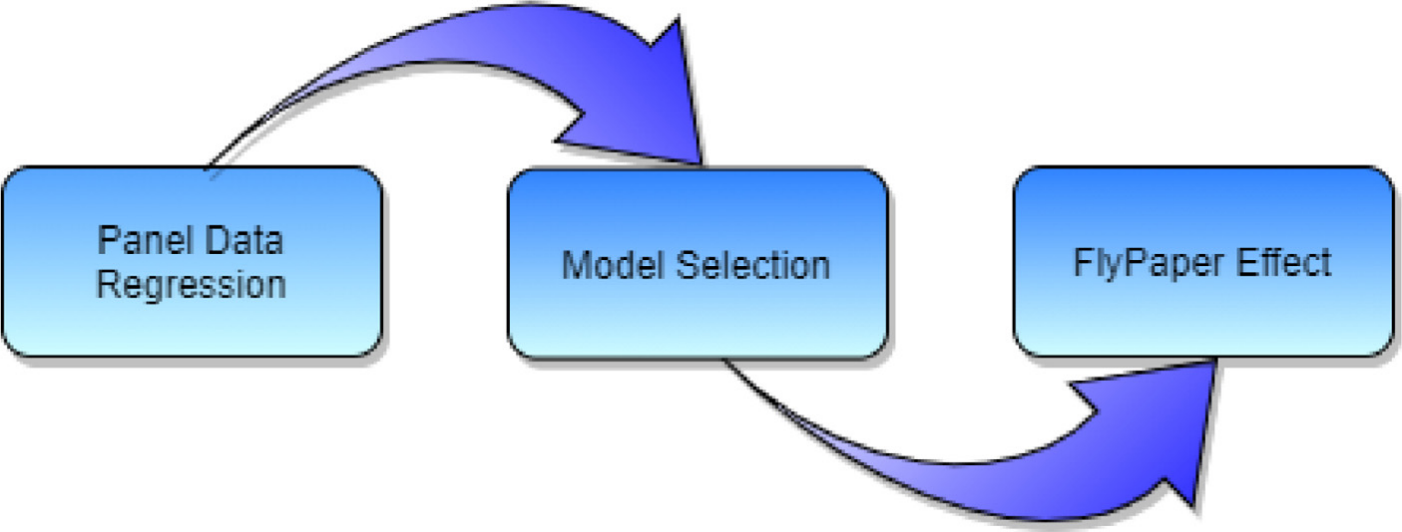
Three main stages in determining the Flypaper Effect on the New Autonomous Regions.

Three main stages were involved to determine flypaper effect namely Data Regression Panel, Model Selection, and Flypaper Effect Determination. Fig. 1 depicts the three phases of this process. The first stage was the Provision of Data Regression Panel for the areas processed by the data. The second stage was to determine the selected model by using several steps. The third stage was the Flypaper Effect determination based on the model chosen.

**Fig. 2:**
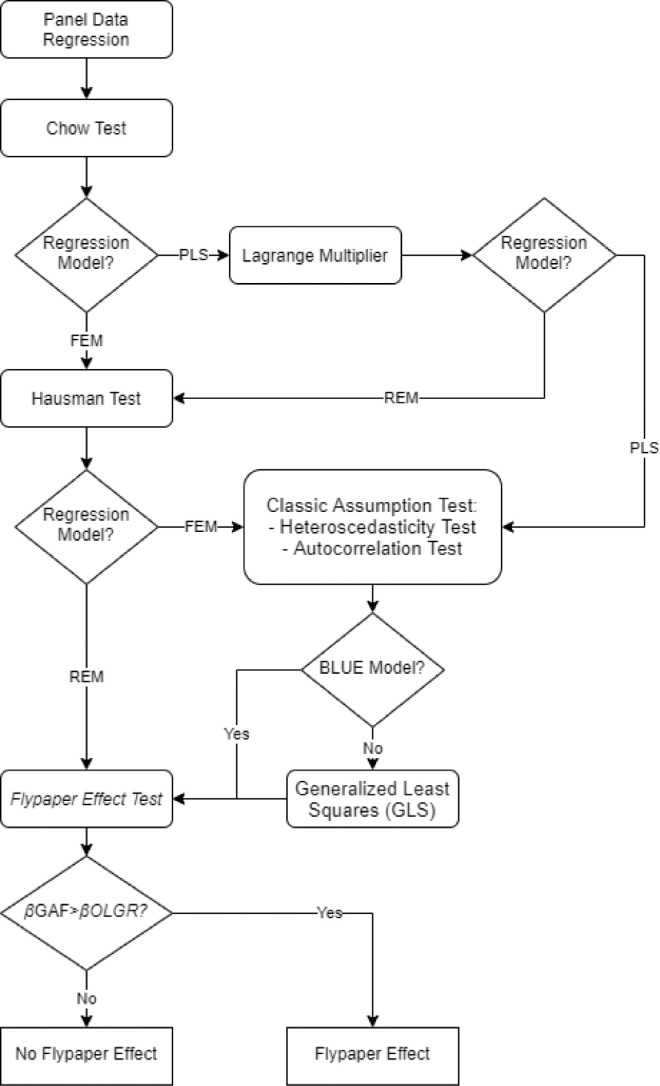
Method for selecting a model that satisfies Best Linear Unavailable Estimator (BLUE).

Fig. 2 depicts the detailed stages in determining the Flypaper Effect and framework method. The Panel Data Regression was the foundation of this process. Several alternative models were selected by several possible tests, including the Chow Test [14], Lagrange Multiplier Test [15], and Hausman Test [16]. The Classic Assumption Test was conducted to select the FEM or PLS model, namely the Heteroscedasticity Test [17] and an Autocorrelation Test [18].

Furthermore, the Generalized Least Squares (GLS) method was used [19] for the models that have not yet satisfied the BLUE condition. After obtaining the most appropriate model for the available panel data, the next step was to determine the Flypaper Effect. At this stage, the process determined whether an area was included in the Flypaper Effect category or not.

## Research design and data processing

This study utilizes standard terms used by the international community or their English translations. To explain the terms similarities that exist in Indonesia and international realms, the following terms are used in both Indonesian and English; Belanja pemerintah daerah as Local Government Expenditure (LGE) [20,21]. Dana Alokasi Umum as the General Allocation Fund (GAF) [22]. Pendapatan Asli Daerah (PAD) as Original Local Government Revenue (OLGR) [23]. Derajat otonomi fiskal (DOF) as Degree of Fiscal Autonomy (DFA).

Hypothesis testing was carried out first on all selected samples and divided into two regional groups: high DFA and low DFA. The division's basis was the degree of fiscal autonomy (DAF) in the New Autonomous Regions (NAR). The DFA ratio was calculated by dividing the local revenue of each NAR by the total revenue of the region concerned. DFA ratios below the average were categorized as low DFA areas, and DFA ratios above-average were classified as high DFA areas [24,25].

This study used descriptive and associative methods; descriptive methods were described as LGE, GAF, and OLGR. The scope of the study was studyingthe New Autonomous Regions during 1999–2021 [8]. The associative process was used to calculate the effect of GAF and OLGR on regional spending. Consequently, the results determine the occurrence of the Flypaper Effect [7].

The data used in this study were secondary ones. namely the General Allocation Funds, local revenue, regional government revenue, and local government spending for 210 New Autonomous Regions in Indonesia. Furthermore, the financial data were assessed from 2016 to 2019. This data source was taken from the Directorate General of Balance, Ministry of Finance Republik of Indonesia [8].

In this study, the statistical calculation process of the data used the STATA version 16 application. STATA is a multipurpose statistical software package developed by StataCorp.

## Samples

The samples comprisede 210 New Autonomous Regions during 1999–2021, consisting of 176 regencies and 34 cities. The sample size was calculated using the formula derived by Slovin with a precision level of 0.05 to obtain a sample size of 138 districts and cities [26]. Sampling was carried out using cluster random sampling technique with allocation allocations and selected 116 regions and 22 cities.

## Operational definitions and scale of variables

This study consisted of three variables, namely LGE, GAF, and OLGR. Each variable was defined, as shown in Table 1. The dependent variable was the variable influenced by the independent variables, whereas the independent variable is the variable that affects the dependent variable.

**Table 1:** Definition of operational variables.

Variable	Scale	Type
Local Government Expenditure (LGE)	Interval	Dependent
General Allocation Fund (GAF)	Interval	Independent
Original Local Government Revenue (OLGR)	Interval	Independent

## Analysis method

### Chow test

The initial stage of the selection was to carry out the Chow test. On the condition that the probability value F is smaller than the significant level (ă=0,05), rejected H0, accepted H1. Conversely, if the probability value of F is greater than the significant level, accepted H0 rejected H1, the hypothesis is as follows:

H_0_: PLS chosen

H_1_: FEM chosen

### Lagrange Multiplier test

Lagrange Multiplier (LM) test provided that if the probability value of chibar2 is smaller than the significant level (ă= 0,05), reject H0, accept H1. Conversely, if the probability value of chibar2 is greater than the significant level, accept H0 reject H1. The hypothesis is as follows:

H_0_: PLS chosen

H_1_: REM chosen

### Hausman test

Furthermore, the Hausman test showed that if the probability value of chi2 is smaller than the significant level (ă=0,05), reject H0 and accept H1, Conversely, if the probability value of chi2 is greater than the significant level, accept H0 and reject H1, and the hypothesis is as follows:

H_0_: REM chosen

H_1_: FEM chosen

### Classic Assumption test

The Classic Assumption Test was used to select the model (set), especially between FEM or PLS. The steps taken in the Classic Assumption Test include processing the data through the Heterocedascity Test calculation process [27] using Koenker-Bassett Test [28] and Autocorrelation Test [29].

Heteroscedasticity test provisions view the data from the probability value t and the level of significance. The significant level is ă, and value is 0.05. If t is greater than the significant level, there is no heteroscedasticity.

After performing the Heteroscedasticity test, the Autocorrelation Test will be performed as follows. The parameters used In the Autocorrelation Test are probability z and a significance level (ă = 0.05). If the probability value is greater than the significance level, then no autocorrelation occurs, or the assumption of non-autocorrelation fulfills. The results of Heteroscedasticity and autocorrelation tests determine whether the Panel Data Regression is BLUE or not.

### Generalized Least Squares (GLS)

GLS is a process to solve the BLUE problem in Panel Data Regression, especially after the Autocorrelation process is done. The technique used through GLS is to estimate unknown parameters in the linear regression model, especially when there is a certain level of correlation between residues in the regression model.

### Flypaper Effect test

The Flypaper Effect hypothesis testing was carried out by comparing the GAF regression coefficient (βGAF) on local government Expenditure with the regression coefficient (βOLGR) on Local Government Expenditure (LGE). If βGAF is greater than βOLGR, then a Flypaper Effect has occurred.

(1) $$LGEit=\beta_{1i}+\beta_{2}GAF_{it}+\beta_{3}OLGR_{it}+U_{it}$$

The regression equation processed is in Eq. (1). Where LGE = Local Government Expenditure, *i* = District / city specific index, *t* = Index for the year, β_1_ = Constanta, β_2, 3_ = Multiple regression coefficients for each independent variable, GAF = General Allocation Fund, OLGR = Original Local Governtment Revenue, and *U* = error.

## Method validation

The determination of process was tested on three sample groups: all samples, low DFA, and high DFA. The three sample groups were used as Panel Data Regression.

**Fig. 3:**
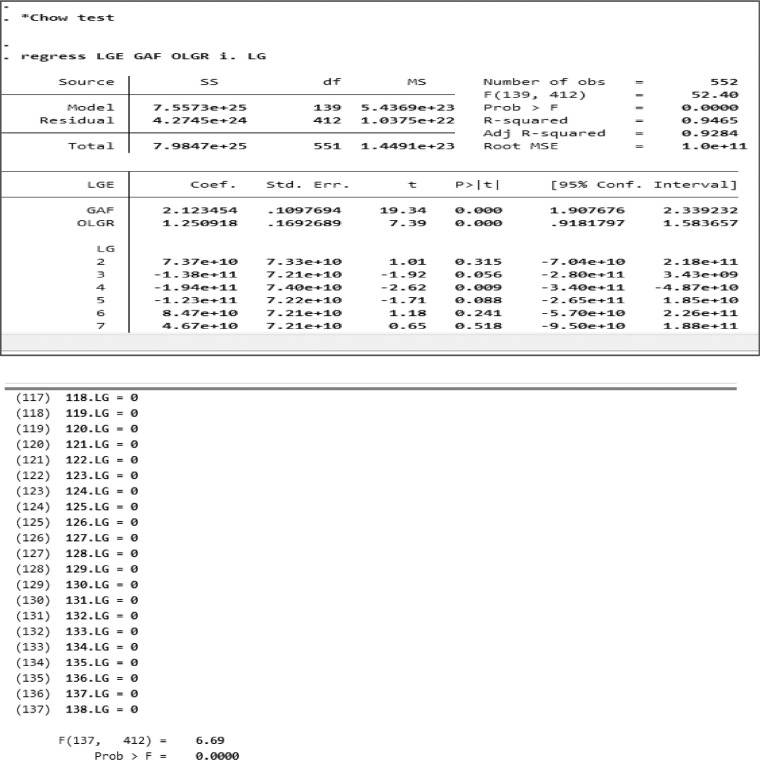
Chow Test using STATA on all samples.

The first test was the Chow Test. Fig. 3 depicted an example of a screenshot when executing the Chow Test calculation using STATA for all. Table 2 shows the results of the three sample groups. The results of the Chow Test on FEM and PLS showed that the selected model was FEM.

**Table 2:** Chow test result.

Group	Probability Value	Decision
All sample	*F* = 0,0000	FEM is selected
Low DFA	*F* = 0,0000	FEM is selected
High DFA	*F* = 0,0000	FEM is selected

DFA: Degree of Fiscal Authonomy, F: The degrees of freedom in the *F*-test.

Table 2 showed the probability value *F* = 0.000 was smaller than the significance level ă = 0.05 in both samples, in the low DFA sample and the high DFA sample. Therefore, H1 was accepted, in this case, the FEM model.

**Fig. 4:**
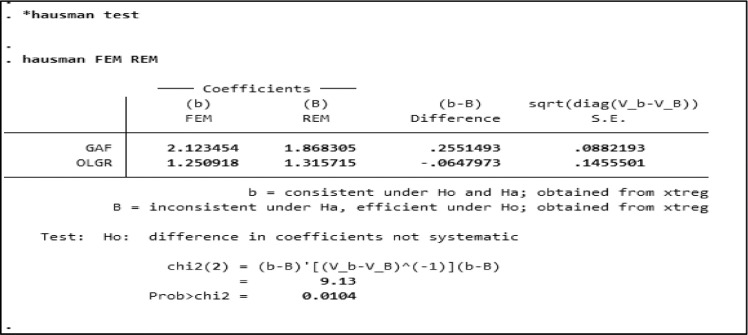
Hausman Test using STATA on the Total Sample.

In this case FEM was the model chosen. Next, Hausman Test was conducted. The Hausman test is to determine between FEM and REM models. An example of the Hausman test's calculation results, especially for all samples using STATA is in Fig. 4. Table 3 the results of the Hausman Test for the three sample groups. The Hausman test resulted in the choice set to be FEM.In this case, FEM model was chosen.

**Table 3:** Hausman test result.

Group	Probability Value	Decision
All Sample	Chi2 = 0,0104	FEM is selected
Low DFA	Chi2 = 0,0393	FEM is selected
High DFA	Chi2 = 0,0085	FEM is selected

DFA: Degree of Fiscal Authonomy, Chi2: The chi-square.

Table 3 showed the chi-square (Chi2) distribution was used in the Hausman test statistics to verify the null hypothesis. The probability value of Chi2 = 0.0104 for the whole sample, Chi2 = 0.0393 for low DFA, and Chi2 = 0.0085 for high DFA. in the case of the FEM model, everything was smaller than the signification level ă = 0.05, then the decision H1 was accepted. The next step was to conduct the Classic Assumption Test.

**Fig. 5:**
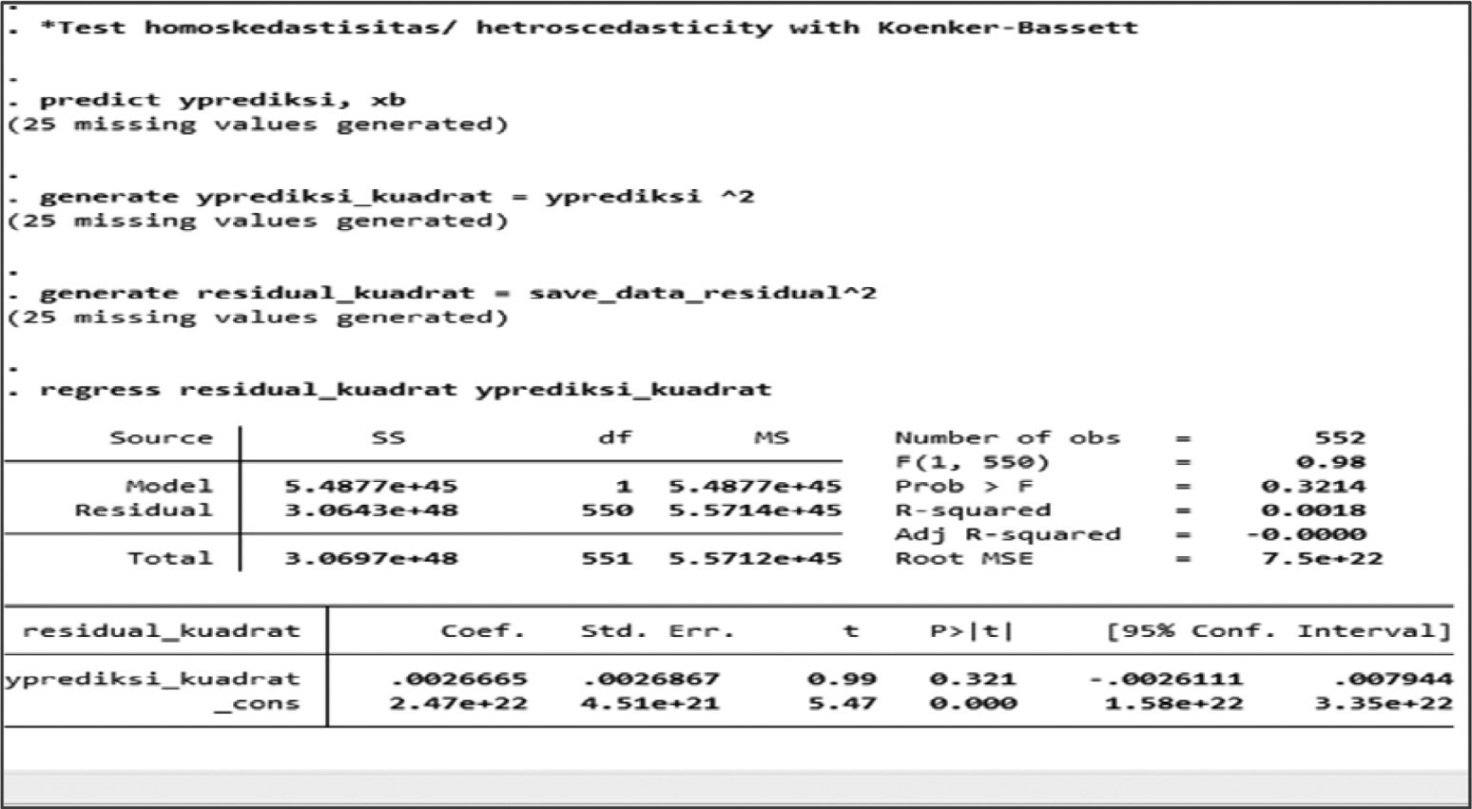
Heteroscedasticity results through STATA for all samples.

In the Classic Assumption Test, the heteroscedasticity test was the first test to be performed. The heteroscedasticity test results for all samples based on STATA are shown in Fig. 5, whereas Table 4 depicts the three sample groups' results.

**Table 4:** Heteroscedasticity test result.

Group	Probability Value	Decision
All Sample	*t* = 0,321	Not occur
Low DFA	*t* = 0,145	Not occur
High DFA	*t* = 0,422	Not occur

DFA: Degree of Fiscal Authonomy, t: The *t*-test result.

Table 4 showed the premise behind heteroscedastic *t*-tests was the variances between two sample data ranges. The probability value *t* = 0.321 for the whole sample. *t* = 0.145 for the low DFA sample, and *t* = 0.422 for the high DFA sample, all greater than the signification level of ă = 0.05. Then, heteroscedasticity did not occur. Based on the heteroscedasticity test, all Panel Data Regression results were free: all samples, high DFA, and low DFA.

**Fig. 6:**
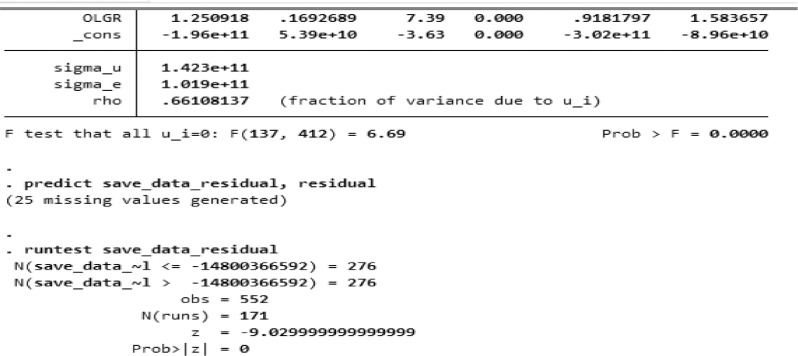
Autocorrelation Test results using STATA for all samples.

Furthermore, the autocorrelation test (test of autocorrelation was carried out. The autocorrelation test results for all samples based on screenshots using STATA are shown in Fig. 6, whereas Table 5 depicts the three sample groups' summaries.

**Table 5:** Autocorrelation test result.

Group	Probability Value	Decision
All Sample	*z* = 0	There was autocorrelation
Low DFA	*z* = 0	There was autocorrelation
High DFA	*z* = 0	There was autocorrelation

DFA: Degree of Fiscal Authonomy, z: The *z*-test score result.

A *z*-test is a statistical test that determines whether the means of two populations vary. The probability value of z in table 5 above was *z* = 0 which was good for all samples, low DFA samples, and high DFA samples. Compared to the signification level ă = 0.05, the z probability value was smaller than the signification level ă. Therefore, the model contained autocorrelation. Based on the results, autocorrelation problem occurred for all samples, high DFA and low DFA.

**Fig. 7:**
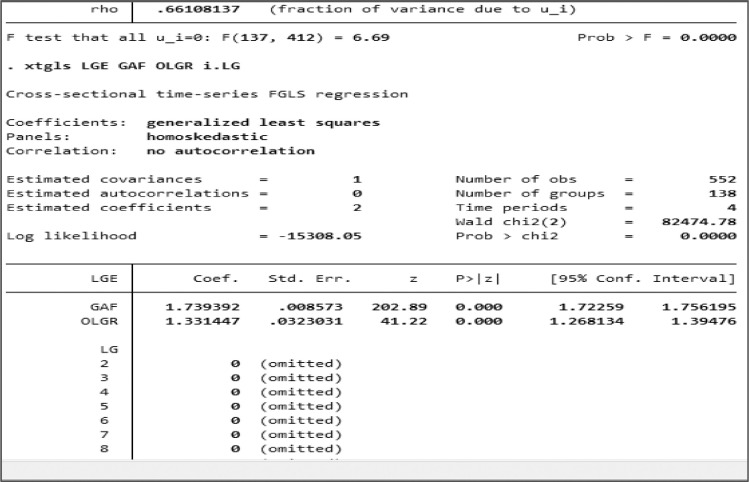
The results of the GLS method for all samples.

To solve the BLUE problem, we used the GLS (Generalized Least Squares) method so that the results showed that autocorrelation did not occur again for all samples, high DFA and low DFA. Fig. 7 depicts The GLS method results for all samples based on screenshots using STATA.

The last one was to test the Flypaper Effect to determine a Flypaper Effect in the three sample groups. The method compared the GAF regression coefficient (βGAF) on Local Government Expenditure with the regression coefficient (βOLGR) on Local Government Expenditure. If the GAF regression coefficient (βGAF) is greater than the regression coefficient (βOLGR), a Flypaper Effect occurs. Otherwise, a Flypaper Effect does not exist.

## Conclusion

Based on the results, the Flypaper Effect assessment method is successfully conducted by using Panel Data Regression. Flypaper Effect analysis is carried out for the New Autonomous Region by using the BLUE model selection method. The process involves the selection of a model, either FEM, REM, or PLS. The models of FEM and PLS, especially, must satisfy the BLUE condition. If it is not BLUE, then the GLS process will be carried out.

Obtaining the Flypaper Effect's condition in the New Autonomous Regions is useful for various purposes, including reviewing the independence of the New Autonomous Regions. In a nutshell, Panel Data Regression is deemed fit to ascertain Flypaper Effect in the New Autonomous Regions (NARs).

## Declaration of Competing Interest

The authors declare that they have no known competing financial interests or personal relationships that could have appeared to influence the work reported in this paper.
